# Association of Online Consumer Reviews of Skilled Nursing Facilities With Patient Rehospitalization Rates

**DOI:** 10.1001/jamanetworkopen.2020.4682

**Published:** 2020-05-14

**Authors:** Kira L. Ryskina, Anietie U. Andy, Kirstin A. Manges, Kierra A. Foley, Rachel M. Werner, Raina M. Merchant

**Affiliations:** 1 Perelman School of Medicine, Division of General Internal Medicine, Department of Medicine, University of Pennsylvania, Philadelphia; 2 Leonard Davis Institute of Health Economics, University of Pennsylvania, Philadelphia; 3 Center for Digital Health, University of Pennsylvania Health System, Philadelphia; 4 University of Pennsylvania, Philadelphia; 5 University of Pennsylvania School of Nursing, Philadelphia; 6 Corporal Michael J. Crescenz VA Medical Center, Philadelphia, Pennsylvania; 7 Perelman School of Medicine, Department of Emergency Medicine, Department of Medicine, University of Pennsylvania, Philadelphia

## Abstract

**Importance:**

There are areas of skilled nursing facility (SNF) experience of importance to the public that are not currently included in public reporting initiatives on SNF quality. Whether patients, hospitals, and payers can leverage the information available from unsolicited online reviews to reduce avoidable rehospitalizations from SNFs is unknown.

**Objectives:**

To assess the association between rehospitalization rates and online ratings of SNFs; to compare the association of rehospitalization with ratings from a review website vs Medicare Nursing Home Compare (NHC) ratings; and to identify specific topics consistently reported in reviews of SNFs with the highest vs lowest rehospitalization rates using natural language processing.

**Design, Setting, and Participants:**

A retrospective cross-sectional study of 1536 SNFs with online reviews on Yelp (a website that allows consumers to rate and review businesses and services, scored on a 1- to 5-star rating scale, with 1 star indicating the lowest rating and 5 stars indicating the highest rating) posted between January 1, 2014, and December 31, 2018. The combined data set included 1536 SNFs with 8548 online reviews, NHC ratings, and readmission rates.

**Main Outcomes and Measures:**

A mean rating from the review website was calculated through the end of each year. Risk-standardized rehospitalization rates were obtained from NHC. Linear regression was used to measure the association between the rehospitalization rate of a SNF and the online ratings. Natural language processing was used to identify topics associated with reviews of SNFs in the top and bottom quintiles of rehospitalization rates.

**Results:**

The 1536 SNFs in the sample had a median of 6 reviews (interquartile range, 3-13 reviews), with a mean (SD) review website rating of 2.7 (1.1). The SNFs with the highest rating on both the review website and NHC had 2.0% lower rehospitalization rates compared with the SNFs with the lowest rating on both websites (21.3%; 95% CI, 20.7%-21.8%; vs 23.3%; 95% CI, 22.7%-24.0%; *P* = .04). Compared with the NHC ratings alone, review website ratings were associated with an additional 0.4% of the variation in rehospitalization rates across SNFs (adjusted *R*^2^ = 0.009 vs adjusted *R*^2^ = 0.013; *P* = .003). Thematic analysis of qualitative comments on the review website for SNFs with high vs low rehospitalization rates identified several areas of importance to the reviewers, such as the quality of physical infrastructure and equipment, staff attitudes and communication with caregivers.

**Conclusions and Relevance:**

Skilled nursing facilities with the best rating on both a review website and NHC had slightly lower rehospitalization rates than SNFs with the best rating on NHC alone. However, there was marked variation in the volume of reviews, and many SNF characteristics were underrepresented. Further refinement of the review process is warranted.

## Key Points

**Question:**

Are reviews of skilled nursing facilities posted on review websites associated with patient rehospitalization rates?

**Findings:**

In this cross-sectional study of 1536 skilled nursing facilities, facilities with the highest rating on 2 review websites had 2.0% lower rehospitalization rates compared with the skilled nursing facilities with the lowest rating. Qualitative comments captured perceptions of experience with skilled nursing facilities not reported elsewhere (eg, quality of physical infrastructure and equipment, staff attitudes and communication with caregivers).

**Meaning:**

These findings suggest that unsolicited online reviews could provide salient information to inform skilled nursing facility selection.

## Teaser

This cross-sectional study of skilled nursing facilities examines the association between rehospitalization rates from skilled nursing facilities and online consumer ratings of those facilities.

## Introduction

More than 40% of patients who require postacute care after hospitalization are discharged to skilled nursing facilities (SNFs), which provide short-term skilled nursing or rehabilitation services after hospital discharge.^1^ One in 4 patients discharged to SNFs for postacute care are rehospitalized within a month.^2^ Rehospitalizations represent a particularly important outcome in this patient population that has been shown to estimate future hospital use,^3^ have negative associations with function^4^ and quality of life.^5^ Hospitals have been subjected to financial pressure to reduce readmissions after facing penalties from Medicare for excessive readmission rates.^6^ In contrast with other postacute care settings, rehospitalizations from SNFs represent an opportune target for hospitals given the hospitals’ ability to influence the choice of SNF. Rehospitalizations from SNFs play a substantial role in the discharging hospital’s readmission rate,^7^ suggesting that hospitals could reduce readmissions by diverting their discharges to SNFs with low readmission rates.

One challenge hospitals and patients face in selecting a SNF is the limited information available on SNF quality and outcomes. Current publicly available ratings of SNFs are not associated with hospital readmissions. Evaluations of the Medicare Nursing Home Compare (NHC) ratings found that a patient’s likelihood of rehospitalization differed by less than 1 percentage point between SNFs with the lowest vs highest star rating.^2,8^ Furthermore, NHC ratings could be used to match more patients with clinically complex conditions to higher-quality SNFs.^9^ Whether higher rehospitalization rates represent an influx of patients who are sicker or worse care quality at a SNF is not observable to consumers. Considering the need for more detailed information on SNF care quality, we sought to assess the association between SNF rehospitalization rates and SNF ratings reported by a large online platform (Yelp, which allows consumers to rate and review businesses and services on a 1- to 5-star rating scale, with 1 star indicating the lowest rating and 5 stars indicating the highest rating).

Unsolicited health-related reviews have been posted on this review website for more than a decade,^10^ with a substantial increase in the past 5 years.^11^ Two advantages of the review website are the short lag time between review submission and posting and the ability to comment on aspects of SNF care that reviewers find salient.^12^ In contrast, NHC ratings are reported with a considerable time lag. Furthermore, HNC does not include a mechanism to relay open-ended information that may be relevant to consumers. The measures are vetted for facilities to know what they are measured on, but this opens possibility for gaming the measures, teaching to the test, and negative spillover on unmeasured aspects of quality.^13,14,15^ Disadvantages of unsolicited online reviews include a lack of transparency regarding the reviewers’ experience with the facility (ie, timing and relationship to the facility), unmeasured selection bias (ie, who posts a review), and intrinsically subjective nature of the ratings.^12,16^

We conducted this national study to evaluate reviews of SNFs on the review website as a potential source of information about SNF quality. We hypothesized that excess rehospitalizations from SNFs may be associated with aspects of quality covered in online reviews. For instance, caregiver perceptions of care may be particularly important in rehospitalizations from SNFs, given that some avoidable rehospitalizations are associated with caregiver demands.^17^ Because the review website reviews are available within days of submission, the review website ratings may more closely track SNF care quality over time. For these reasons, we hypothesized that the review website ratings of SNFs are associated with rehospitalization rates. Our objectives were to: (1) assess the association between rehospitalization rates and the review website ratings of SNFs; (2) compare how well the review website vs NHC ratings explain the variation in rehospitalizations from the SNFs; and (3) identify specific topics consistently reported in reviews of SNFs with the highest vs lowest rehospitalization rates using natural language processing.

## Methods

The study was deemed exempt from review by the University of Pennsylvania Institutional Review Board as not meeting the regulatory definition of human research. The reporting of this study follows the Strengthening the Reporting of Observational Studies in Epidemiology (STROBE) reporting guideline for cross-sectional studies,^18^ and the Standards for Reporting Qualitative Research (SRQR) guideline for qualitative studies.^19^

### Data and Sample

The review website publishes free-text comments submitted online by consumers as well as a cumulative average 5-point rating of all submissions. There is no expiration time frame for older reviews, but more recent reviews appear first on screen. The comments are unedited. The review website requires firsthand experiences from contributors, using proprietary algorithms to highlight reviews that “best reflect the opinions of the Yelp community” and exclude “fraudulent submissions”.^11^ We used Yelp in this study because it provides fraud detection mechanisms that allowed us to remove reviews that were likely fraudulent.^20^

To merge the review website ratings to NHC data, we manually extracted all 16 191 reviews in the nursing home category from the review website, consistent with the terms of service of the platform. The reviews represented 2467 unique business names and addresses and were submitted between August 27, 2005, and January 17, 2019. We collected all available rehospitalization rates on NHC, which were measured between the first quarter of 2014 and last quarter of 2018. There were 14 787 SNFs with available overall rating and rehospitalization rates in NHC. These data were linked to the Medicare Provider of Services files containing SNF characteristics (eg, number of beds and ownership type).

The review website facilities were matched to NHC identifiers via 2 rounds of matching. First, NHC name and the review website business name were considered matched if there was an exact string match between the names and zip codes. Second, the review website facilities not matched in the first round were assigned to an unmatched NHC facility located in the same zip code if the names varied by at most 3 characters. All facilities matched in the second round of matching were checked manually by one of us (K.L.R.) using a prespecified protocol. A match was considered acceptable if facility names differed by an article (ie, a/an/the), space (eg, “Willowbrook” vs “Willow Brook”), abbreviation (“Rehabilitation & Healthcare” vs “Rehabilitation and healthcare”), or generic business designation (eg, “LLC” or “SNF”). Otherwise, a match was considered ambiguous and was deleted (eg, “St Mary” vs “St Martha”). The combined data set included 1536 SNFs with 8548 review website reviews, NHC ratings, and readmission rates measured between January 1, 2014, and December 31, 2018 (10.4% of SNFs with rehospitalization rates and ratings on NHC) (eFigure in the Supplement).

### Variables

The NHC reports an annual overall 5-star rating calculated based on an algorithm that weighs health inspections and staffing relative to quality measures.^21^ In 2016, NHC began to report measures of hospital readmissions as part of the quality measures. We used the risk-standardized 30-day rehospitalization rate for short-term SNF stays calculated using the NHC Quality Measures specifications.^22^ The rate is adjusted for patient demographics, clinical conditions, and functional status using Medicare claims and SNF clinical assessments. The risk-standardized rehospitalization rates are measured over a 12-month period and updated quarterly (lag time of 9 months).^22^ We used the rates measured over the 12-month period from January to December of each year in the study. To match to the corresponding NHC measurement interval for rehospitalization rates, we calculated a mean review website rating through the end of each calendar year of the study.

### Statistical Analysis

The analyses were conducted between January 1 and September 30, 2019. We compared SNF characteristics in our sample with the characteristics of all NHC SNFs during the study period using 1 sample test of proportion and unpaired *t* test for means.^23^ *P* values less than .05 were considered statistically significant; all hypothesis tests were 2-sided.

Analyses were performed at the SNF-year level. To measure the association between rehospitalization rates, NHC overall rating, and review website rating, we estimated 3 sets of models. First, we estimated the risk-standardized rehospitalization rate as a function of the overall NHC star rating. Second, the same models were estimated with the review website ratings instead of NHC ratings. Third, the models were estimated with both the review website rating and NHC rating. Linear probability models were used to estimate the rates. The difference in the *R*^2^ statistic between the first, second, and third set of models, adjusted for the number of independent variables in each model, was used to measure the proportion of the variance in readmission rates associated with the review website variables. To do so, we first calculated the adjusted *R*^2^ statistic for each model, which measures the proportion of variance associated with variables in the model. Next, we subtracted the adjusted *R*^2^ for the NHC only model from the *R*^2^ for the combined NHC and review website ratings model. The difference represents the proportion of variance associated with review website ratings, in addition to the proportion of variance associated with the NHC ratings alone. The Huber-White sandwich estimator was used in all regressions to account for clustering of observations within SNFs.^24^ Statistical analyses were performed using Stata, version 15 (StataCorp LP).

To generate topics (word clouds), which consist of clusters of co-occurring words, we extracted the following features from each review: the relative frequency of single words and phrases with at most 3 words, and the distribution of 20 latent Dirichlet allocation (LDA) topics associated with the reviews for SNFs in the top vs bottom quintiles of rehospitalization rates. LDA is a statistical generative model commonly used in natural language processing that identifies topics in text based on word co-occurrence.^25^

We performed a number of additional analyses. First, we re-estimated the rehospitalization models on a subsample of SNFs with at least 3 reviews. Second, we re-estimated the models including SNF characteristics that were selected a priori because they were previously found to be associated with SNF quality (region,^26^ rural vs urban location,^27^ size (<100, 100-199, or 200+ beds),^2^ ownership (for-profit vs nonprofit),^28^ presence of advanced practitioners,^29^ whether the facility was part of a chain,^28^ and whether it was hospital based^30^). Third, we performed qualitative thematic analyses of the reviews for SNFs in the top vs bottom quintiles of rehospitalization rates and triangulated the results with the LDA generated word clouds.^31^ Two hundred reviews were randomly selected and independently reviewed by 2 trained qualitative researchers with prior clinical experience working in SNFs (K.M. and K.F.). We used an inductive approach to identify common themes across the reviews. First, the 2 qualitative researchers independently reviewed all comments and coded recurrent topics and ideas. Next, the researchers conducted a series of group discussions to go over the topics their identified. Through these iterative discussions, a consensus list of most salient topics was developed. Lastly, the researchers triangulated the findings with the content of the word clouds. We used the DLATK package for the LDA analysis and the word clouds.^32^

## Results

### Study Sample

The characteristics of SNFs in the main sample, in the subsample of SNFs with at least 3 reviews, and all SNFs with rehospitalization rates reported during the study period are shown in Table 1. The SNFs in the main sample had a median of 6 reviews (interquartile range 3-13), with a mean (SD) review website rating of 2.7 (1.1). The mean (SD) rehospitalization rate was 22.5% (5.1). Compared with all SNFs on NHC, SNFs located in the West, in urban areas, large in size, and with for-profit ownership were over-represented in the main study sample. The subsample of 1011 SNFs with at least 3 reviews was similar to the main sample (Table 1).

**Table 1.** Baseline Characteristics of SNFs, 2014-2018

Characteristic	SNFs, %		
	All NHC (n = 14 787)^a^	In the main review website sample (n = 1536)^b^	In the subsample with ≥3 review website reviews (n = 1011)
Region			
Northeast	18.0	15.9^c^	14.1^c^
South	36.5	20.2^c^	18.5^c^
Midwest	31.1	14.5^c^	12.3^c^
West	14.4	50.2^c^	55.1^c^
Size, beds (No.)			
Small (<100)	44.3	39.3^c^	40.3^c^
Medium (100-199)	49.0	50.4	49.0
Large (≥200)	6.7	10.3^c^	10.7^c^
Ownership			
Nonprofit	34.9	23.6^c^	21.9^c^
For-profit	65.2	76.4^c^	78.1^c^
Location			
Urban	73.5	96.8^c^	96.1^c^
Rural	26.5	3.2^c^	3.9^c^
Any advanced practitioners (eg, NPs)	50.9	53.7^b^	51.8
Part of a chain	58.6	60.3	59.3
Hospital-based	3.9	1.5^c^	1.4^c^
NHC rating			
1 star	13.6	7.6^c^	6.4^c^
2 stars	19.9	17.2^c^	17.0^c^
3 stars	17.8	15.7^c^	15.3^c^
4 stars	23.4	25.5	25.6
5 stars	25.3	34.3^c^	35.7^c^
Review website			
Rating, mean (SD)	NA	2.7 (1.1)	2.8 (1.0)
Reviews per SNF, median (IQR)	NA	6 (3-13)	8 (4-16)
Rehospitalization rate per 100 discharges, mean (SD)	21.8 (6.0)	22.5 (5.1)^c^	22.5 (5.0)^c^

Abbreviations: IQR, interquartile range; NA; not applicable; NHC, Nursing Home Compare; NP, nurse practitioner; SNF, skilled nursing facility.

^a^ All SNFs in NHC with star ratings and rehospitalization rates reported in 2014-2018.

^b^ The review website, Yelp, is an online platform that allows consumers to rate and review businesses and services; scored on a 1- to 5-star rating scale, with 1 star indicating the lowest rating and 5 stars indicating the highest rating.

^c^ Different from population (all NHC SNFs) at *P* < .05.

### Review Website Ratings, NHC Ratings, and Rehospitalization Rates

Of the 3614 SNF ratings during 2014-2018, over a third (34.3%) of NHC ratings were 5-star and 7.6% were 1-star (Table 2). In contrast, 12.1% of review website ratings were in the highest (>4.4) and 22.1% were in the lowest category (<1.5). The 2 sets of ratings matched (eg, 1-star rating on NHC and <1.5 points on the review website) in 20.7% of cases.

**Table 2.** Risk-Standardized 30-Day Hospital Readmission Rates From SNF^a^

Variable	SNF-years, No. (%)^b^	Readmission rate, % (95% CI)		
		Models with NHC rating	Models with review website rating	Models with both NHC and review website ratings
**Nursing Home Compare star rating (n = 3614)**				
1-star (worst)	276 (7.6)	23.4 (22.8-23.9)	NA	23.3 (22.7-23.9)
2-star	621 (17.2)	22.8 (22.4-23.2)	NA	22.7 (22.3-23.1)
3-star	567 (15.7)	22.7 (22.3-23.1)	NA	22.7 (22.3-23.1)
4-star	909 (25.2)	22.7 (22.4-23.1)	NA	22.7 (22.4-23.1)
5-star (best)	1241 (34.3)	21.8 (21.5-22.1)^c^	NA	21.8 (21.6-22.1)^c^
**Review website rating (n = 3614)**				
<1.5 (worst)	798 (22.1)	NA	22.8 (22.4-23.2)	22.7 (22.3-23.0)
1.5-2.4	770 (21.3)	NA	22.8 (22.5-23.2)	22.8 (22.4-23.1)
2.5-3.4	980 (27.1)	NA	22.5 (22.2-22.8)	22.5 (22.2-22.8)
3.5-4.4	628 (17.4)	NA	21.9 (21.5-22.4)^c^	22.1 (21.7-22.5)
>4.4 (best)	438 (12.1)	NA	21.9 (21.4-22.4)^d^	22.0 (21.5-22.5)^e^
**NHC and review website ratings (n = 747)**				
1-star and <1.5 review website rating (worst)	101 (2.8)	NA	NA	23.3 (22.7-24.0)
2-star and 1.5-2.4 review website rating	146 (4.0)	NA	NA	23.1 (22.6-23.6)
3-star and 2.5-3.4 review website rating	148 (4.1)	NA	NA	22.8 (22.3-23.3)
4-star and 3.5-4.4 review website rating	164 (4.5)	NA	NA	22.4 (21.9-22.9)
5-star and >4.4 review website rating (best)	188 (5.2)	NA	NA	21.3 (20.7-21.8)^f^
Adjusted *R*^2^	NA	0.009	0.007	0.013^g^

Abbreviations: NA, not applicable; NHC, Nursing Home Compare; SNF, skilled nursing facilities.

^a^ The review website, Yelp, is an online platform that allows consumers to rate and review businesses and services; scored on a 1- to 5-star rating scale, with 1 star indicating the lowest rating and 5 stars indicating the highest rating.

^b^ SNF-years.

^c^ Significantly different from the reference group (lowest rating) *P* < .001.

^d^ Significantly different from the reference group (lowest rating) *P* = .008.

^e^ Significantly different from the reference group (lowest rating) *P* = .004.

^f^ Significantly different from the reference group (lowest rating) *P* = .04.

^g^ Significantly different from Nursing Home Compare Rating only model, *P* = .003.

SNFs with the highest NHC rating (5-star) had 1.6% lower rehospitalization rate compared with SNFs with the lowest rating (1-star) (21.8%; 95% CI, 21.5% to 22.1% for 5-star vs 23.4%; 95% CI, 22.8% to 23.9% for 1-star; *P* < .001) (Table 2). SNFs with the highest review website rating (≥4.5 out of 5) had 0.9% lower rehospitalization rate compared with SNFs with the lowest review website rating (<1.5 out of 5) (21.9%; 95% CI, 21.4% to 22.4% for the review website rating >4.4 stars vs 22.8%; 95% CI, 22.4% to 23.2% for the review website rating <1.5 stars; *P* = .008). Facilities with the best rating on both the review website and NHC had 2.0% lower rehospitalization rates compared with the SNFs with the worst rating on both websites (21.3%; 95% CI, 20.7% to 21.8% for SNFs with the highest ratings on both websites vs 23.3%; 95% CI, 22.7% to 24.0% for SNFs with the lowest ratings on both websites, *P* = .04).

### Assessment of Review Website vs NHC Ratings

NHC ratings alone and review website ratings alone were associated with approximately between 0.7% and 0.9% of variance across rehospitalization rates from SNFs (adjusted *R*^2^ = 0.009 for the NHC only model and adjusted *R*^2^ = 0.007 for the review website only model). Both the review website and NHC ratings together increased the proportion of variance in rehospitalization rates from SNFs by 0.4% from 0.9% to 1.3% (adjusted *R*^2^ = 0.009 vs adjusted *R*^2^ = 0.013; *P* = .003) (Table 2).

We identified 5 topics representing differences in reviews between SNFs with high vs low rehospitalization rates: environment (eg, facility atmosphere and experiences in the physical space), staff attitudes and behaviors (eg, affect, professionalism, and caring behaviors), management (eg, billing, customer service, and organizational culture), communication (eg, effectiveness of transferring information among staff and caregivers), and care quality (eg, delivery of safe care, managing pain, and performing activities of daily living) (Table 3).

**Table 3.** Common Themes From Reviews of SNFs With High vs Low Rehospitalization Rates

Theme	Theme defined	Language from word clouds	Rehospitalization rates	
			High	Low
Communication	Staff ability to transfer information among facility team, with caregivers, and with external providers	Called; asked; speak; told; phone; doctor; nurse; back; finally	“It takes over 20 minutes or more for someone to attend to a patient when they hit their call button.” (1958)	“ I must say I am impressed with staff...There are people constantly checking on my dad... Between the doctor’s nurse practitioner nursing and therapy staff they got his pain under control. They kept me in the loop. [I] can’t thank [them] enough.” (839)
Environment	Experiences in the physical space and general atmosphere of the facility	Home; life; activities; months; years; living; feel; friends	“Upon arrival I was shown to her room. It had garbage on the floor.” (16)	“The facility itself is very accessible, clean, newer, bright, inviting, and has state-of-the-art equipment for the level of rehab we needed.” (635)
Staff attitudes and behaviors	Responses to staff members affect, display of professionalism, and caring behaviors	Nursing; family; teamwork; great; experience; hard work; caring	“They just like rich people. If you ask question about meds they get upset. So rude.” (1028)	“The staff are caring individuals who truly put patients and their family first. They are a friendly team who work well together and provide great care.” (554)
Management	Issues around billing, customer service, and organizational culture	Insurance; information; money; customer service; office	“Horrible customer service. Called for pricing and questions they are rude and literally will hang up on you. I guess I won't be using them. They lost my money.” (667)	“The management is so friendly, they are like family.” (34)
Care quality	Delivery of safe care, especially performing activities of daily living (ie, patient hygiene), managing pain, and preventing adverse events	Hospital; surgery; doctor; pain; infection; medications; blood	“My dad was becoming worse and had a high temperature 105. I called and spoke with his nurse about my concerns. She said his vital signs were good and he responded to her. I demanded they call an ambulance and transfer him to the hospital…He was unable to speak temp 105 had pneumonia dehydrated and had MRSA.” (1212)	“Great facility very caring people work here physical therapy was great husband like the food bed was comfortable and when he called for help they came quickly. I highly recommend after surgery! My husband had a complete knee replacement and overall care was fabulous!!” (388)

Abbreviation: SNF, skilled nursing facilities.

The findings for the subsample of SNFs with at least 3 reviews were generally consistent with the main results (eTable 1 in the Supplement). The association between online ratings and rehospitalization rates were not sensitive to adjustment for SNF characteristics (eTable 2 in the Supplement).

## Discussion

Online consumer ratings of SNFs published on the review website provided novel information associated with a small albeit significant proportion of variation in SNF rehospitalization rates. The use of the review website and NHC ratings together identified SNFs with slightly lower rehospitalization rates compared with NHC ratings alone. The review website reviews identified several areas of importance to the consumers, such as the quality of physical infrastructure and equipment, staff attitudes, and communication with caregivers.

In a recent survey, hospitals reported that forming preferred provider networks of SNFs in their area represented a leading strategy for adjusting to alternative purchasing reforms in postacute care.^33^ Little information is available to date about how hospitals select SNFs into their network. Since 2016, NHC reported rehospitalization rates as part of its SNF quality measures, but the rates are reported with considerable lag time. Hospitals may be able to track internal readmission rates in a more timely manner, but those rates would not reflect readmissions to other hospitals. Our findings suggest that online reviews could inform the selection process by providing additional information to help identify SNFs with the lowest readmission rates. However, absolute differences in readmission rates between the highest vs lowest-rated SNFs were small, and we did not observe significant differences in readmissions between SNFs in the middle categories.

Consistent with prior studies,^34,35,36^ several topics regularly mentioned in the review website reviews are not currently covered by NHC, including staff attitudes and communication as well as physical environment (cleanliness and comfort). Specifically, reviewers commented about caring, helpful, and professional staff and fresh food, nice meals, and private rooms for SNFs with low readmission rates. For SNFs with high readmission rates, reviewers commented about waiting for call back, lack of choice and autonomy, and poor administration and billing. NHC may consider incorporating systematic measures of these aspects of patient and caregiver experience into SNF ratings.

Our findings also highlight previously noted concerns with online information about health care facilities. We were unable to obtain demographic information about the review website reviewers or the nature of their experience with the facilities (eg, timing and duration of stay, relationship to the patient). Sugarman et al^37^ found that online information about SNFs is not well accessible (eg, written in college-level language), and does not contain information of interest to likely consumers (eg, quality of meals). Recent evaluations of consumer experience with NHC reported limited awareness of the website and suspicion about the quality of information available.^38,39^ Whether unsolicited online reviews are associated with consumer decisions or their likelihood to be satisfied with their SNF choice depends on the success of efforts to address these concerns, such as generating ratings in way that is more representative, protects privacy, and remains timely.

### Limitations

This study has several limitations. First, only 10% of the SNFs had any reviews and 34% of those had fewer than 3 reviews. However, the findings from the subsample of SNFs with 3 or more reviews were consistent with the main results. Second, we were not able to distinguish between appropriate and excessive rehospitalizations, or rehospitalizations specifically because of poor quality of care in the SNF. For example, higher readmission rates to a hospital from a SNF may be due to the hospital discharging patients prematurely and may not reflect the quality of care in the SNF. Third, the SNFs with the review website reviews were not representative of all US SNFs. For example, almost all the SNFs in the sample were located in urban areas and half were located in the west. Our findings suggest that certain biases persist in online reviews (eg, computer access in urban vs rural areas, age). Efforts to address these concerns could start with improving transparency of fraud detection algorithms and sharing characteristics of online reviewers.

## Conclusions

To our knowledge, this is the largest evaluation of unsolicited online reviews of SNFs to date. Consistent with prior work, we identified several domains of SNF experience that are not currently covered by NHC. Furthermore, SNFs with the best rating on both the review website and NHC had slightly lower rehospitalization rates than SNFs with the best rating on NHC alone. However, there was marked variation in the volume of reviews and many SNF characteristics were under-represented. These findings suggest that, if key limitations are addressed, unsolicited online reviews could provide salient information to inform SNF selection.

## Notes

Supplement. **eTable 1.** Risk-Standardized 30-Day Hospital Readmission Rates from SNF by SNF Yelp and Nursing Home Compare Ratings: Sub-sample of SNFs with at Least Three Reviews **eTable 2.** Risk-Standardized 30-Day Hospital Readmission Rates from SNF by SNF Yelp and Nursing Home Compare Ratings: Main Study Sample, Models Adjusted for SNF Characteristics **eFigure.** Sample Selection
