# Supplementary material for: Association of Online Consumer Reviews of Skilled Nursing Facilities With Patient Rehospitalization Rates
Source: JAMA Netw Open. 2020 May 14;3(5):e204682. doi: 10.1001/jamanetworkopen.2020.4682 (PMC7225899; doi:10.1001/jamanetworkopen.2020.4682)
Supplement: Supplement. — eTable 1. Risk-Standardized 30-Day Hospital Readmission Rates from SNF by SNF Yelp and Nursing Home Compare Ratings: Sub-sample of SNFs with at Least Three Reviews eTable 2. Risk-Standardized 30-Day Hospital Readmission Rates from SNF by SNF Yelp and Nursing Home Compare Ratings: Main Study Sample, Models Adjusted for SNF Characteristics eFigure. Sample Selection [file jamanetwopen-3-e204682-s001.pdf]

Supplementary Online Content

Ryskina KL, Andy AU, Manges KA, Foley KA, Werner RM, Merchant RM. Association of online consumer reviews of skilled nursing facilities with patient rehospitalization rates. *JAMA Netw Open*. 2020;3(5):e204682. doi:10.1001/jamanetworkopen.2020.4682

eTable 1. Risk-Standardized 30-Day Hospital Readmission Rates from SNF by SNF Yelp and Nursing Home Compare Ratings: Sub-sample of SNFs with at Least Three Reviews

eTable 2. Risk-Standardized 30-Day Hospital Readmission Rates from SNF by SNF Yelp and Nursing Home Compare Ratings: Main Study Sample, Models Adjusted for SNF Characteristics

eFigure. Sample Selection

This supplementary material has been provided by the authors to give readers additional information about their work.

© 2020 Ryskina KL et al. *JAMA Network Open.*

eTable 1. Risk-Standardized 30-Day Hospital Readmission Rates from SNF by SNF Yelp and Nursing Home Compare Ratings: Sub-sample of SNFs with Three or More Reviews

	**Models with NHC Ratings**		**Models with Yelp Ratings**		**Models with both NHC and Yelp Ratings**	
	Readmission Rate, %	95% CI	Readmission Rate, %	95% CI	Readmission Rate, %	95% CI
**Nursing Home Compare Star Rating**						
1-star (Worst)	23.6	23.0-24.3	-	-	23.5	22.9-24.2
2-star	22.9	22.5-23.4	-	-	22.9	22.5-23.4
3-star	22.7*	22.3-23.2	-	-	22.7	22.2-23.1
4-star	22.8*	22.5-23.2	-	-	22.8	22.5-23.2
5-star (Best)	21.8*	21.5-22.1	-	-	22.0	21.7-22.3
**Yelp rating**						
<1.5 (Worst)	-	-	22.7	22.3-23.2	22.9	22.5-23.4
1.5-2.4	-	-	23.0	22.6-23.3	22.9	22.6-23.3
2.5-3.4	-	-	22.6	22.3-22.9	22.6	22.2-22.9
3.5-4.4	-	-	22.0*	21.6-22.4	22.1	21.6-22.5
>4.4 (Best)	-	-	21.7*	21.1-22.3	22.0	21.4-22.6
**NHC and Yelp Ratings**						
1-star & <1.5 Yelp rating (Worst)	-	-	-	-	22.9	21.6-24.2
2-star & 1.5-2.4 Yelp rating	-	-	-	-	22.6	21.8-23.4
3-star & 2.5-3.4 Yelp rating	-	-	-	-	22.8	21.9-23.7
4-star & 3.5-4.4 Yelp rating	-	-	-	-	22.9	22.1-23.8
5-star & >4.4 Yelp rating (Best)	-	-	-	-	22.0	20.1-21.9
Adjusted R^2	0.012		0.006		0.020#	

Note: SNF, skilled nursing facility; NHC, Nursing Home Compare
* Significantly different from the reference group (lowest rating) at p<0.05
# Significantly different from Nursing Home Compare Rating only model at p<0.05

© 2020 Ryskina KL et al. *JAMA Network Open.*

eTable 2. Risk-Standardized 30-Day Hospital Readmission Rates from SNF by SNF Yelp and Nursing Home Compare Ratings: Main Study Sample, Models with SNF Characteristics

	Models with NHC Rating		Models with Yelp Rating		Models with both NHC and Yelp Ratings	
	Readmission Rate, %	95% CI	Readmission Rate, %	95% CI	Readmission Rate, %	95% CI
**Nursing Home Compare Star Rating**						
1-star (Worst)	23.2	22.7-23.8	-	-	23.3	22.7-23.8
2-star	22.7	22.3-23.1	-	-	22.8	22.3-23.2
3-star	22.7	22.3-23.1	-	-	22.6	22.2-23.0
4-star	22.8	22.5-23.1	-	-	22.8	22.5-23.1
5-star (Best)	21.8*	21.5-22.1	-	-	21.8*	21.5-22.1
**Yelp rating**						
<1.5 (Worst)	-	-	22.6	22.2-23.0	22.5	22.1-22.9
1.5-2.4	-	-	22.8	22.4-23.1	22.8	22.4-23.1
2.5-3.4	-	-	22.5	22.2-22.8	22.5	22.2-22.8
3.5-4.4	-	-	22.1	21.7-22.5	22.2	21.8-22.6
>4.4 (Best)	-	-	22.0*	21.6-22.5	22.1*	21.6-22.6
**NHC and Yelp Ratings**						
1-star & <1.5 Yelp rating (Worst)	-	-	-	-	23.3	22.6-23.9
2-star & 1.5-2.4 Yelp rating	-	-	-	-	23.1	22.6-23.6
3-star & 2.5-3.4 Yelp rating	-	-	-	-	22.8	22.3-23.2
4-star & 3.5-4.4 Yelp rating	-	-	-	-	22.5	22.0-23.0
5-star & >4.4 Yelp rating (Best)	-	-	-	-	21.2*	20.7-21.8
**SNF Characteristics**						
Region						
West	21.9	21.6-22.2	21.9	21.6-22.1	21.9	21.6-22.1
Northeast	22.6*	22.2-23.0	22.6*	22.2-23.0	22.6*	22.2-23.0
South	22.9*	22.5-23.3	22.9*	22.6-23.3	22.9*	22.5-23.3
Midwest	23.6*	23.1-24.0	23.6*	23.2-24.1	23.6*	23.2-24.1
(continued on next page)						

Note: SNF, skilled nursing facility; NHC, Nursing Home Compare
* Significantly different from the reference group (lowest rating) at p<0.05
[#] Significantly different from Nursing Home Compare Rating only model at p<0.05

© 2020 Ryskina KL et al. *JAMA Network Open.*

eTable 2 (continued). Risk-Standardized 30-Day Hospital Readmission Rates from SNF by SNF Yelp and Nursing Home Compare Ratings: Main Study Sample, Models with SNF Characteristics

	**Models with NHC Rating**		**Models with Yelp Rating**		**Models with both NHC and Yelp Ratings**	
	Readmission Rate, %	95% CI	Readmission Rate, %	95% CI	Readmission Rate, %	95% CI
Location						
Urban	22.5	22.4-22.7	22.5	22.4-22.7	22.5	22.4-22.7
Rural	19.8*	18.5-21.1	20.0*	18.6-21.3	19.8*	18.5-21.2
Size						
Small (<100 beds)	22.4	21.9-22.9	22.4	21.8-22.9	22.5	21.9-23.0
Medium (100-199 beds)	22.2	21.7-22.6	22.2	21.8-22.7	22.2	21.7-22.6
Large (200 or more beds)	21.7	20.9-22.5	21.8	21.0-22.7	21.7	20.8-22.5
Ownership						
Non profit	21.8	21.4-22.1	21.7	21.3-22.1	21.8	21.4-22.2
For profit	22.7*	22.5-22.9	22.7*	22.5-22.9	22.7*	22.5-22.9
Any advanced practitioners						
No	22.5	22.2-22.6	22.5	22.2-22.7	22.5	22.2-22.7
Yes	22.5	22.2-22.7	22.5	22.2-22.7	22.5	22.2-22.7
Part of a chain						
No	22.1	21.7-22.4	22.0	21.6-22.4	22.1	21.7-22.4
Yes	22.7*	22.5-22.9	22.6*	22.4-22.8	22.6*	22.4-22.8
Hospital-based						
No	22.4	22.3-22.6	22.4	22.3-22.6	22.4	22.3-22.6
Yes	24.0*	22.5-25.5	23.8*	22.3-25.3	23.9	22.4-25.4
Adjusted R^2	0.033		0.033		0.040[#]	

Note: SNF, skilled nursing facility; NHC, Nursing Home Compare
* Significantly different from the reference group at p<0.05
[#] Significantly different from Nursing Home Compare Rating only model at p<0.05

© 2020 Ryskina KL et al. *JAMA Network Open.*

eFigure. Sample Selection

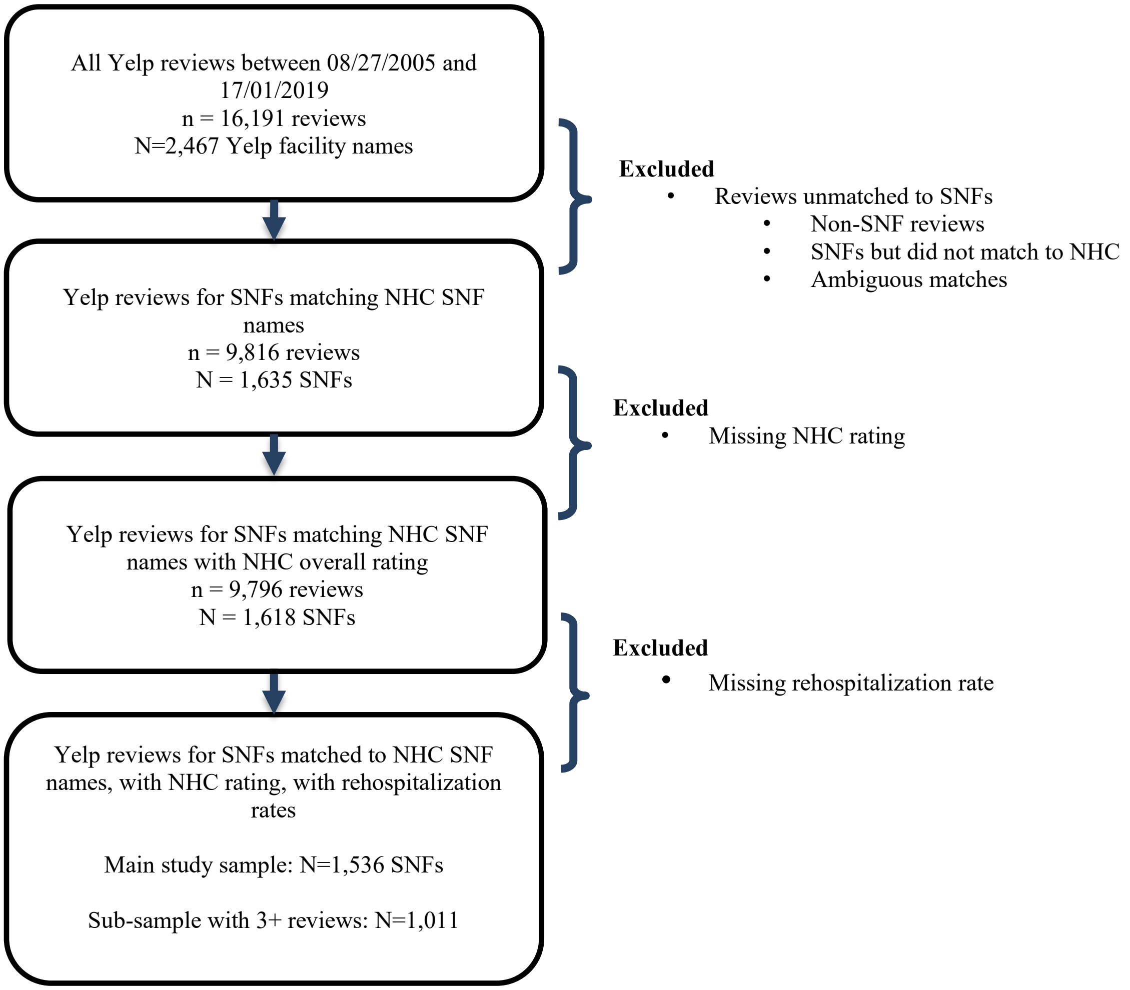

Note: SNF, skilled nursing facility; NHC, Nursing Home Compare

© 2020 Ryskina KL et al. *JAMA Network Open.*
